# Expression of SLC4A11 protein in mouse and rat medulla: a candidate transporter involved in outer medullary ammonia recycling

**DOI:** 10.14814/phy2.14089

**Published:** 2019-05-23

**Authors:** Michael T. Gee, Ira Kurtz, Thomas L. Pannabecker

**Affiliations:** ^1^ Department of Physiology Banner‐University Medical Center University of Arizona Tucson AZ 85724; ^2^ Division of Nephrology David Geffen School of Medicine University of California, Los Angeles (UCLA) Los Angeles CA; ^3^ Brain Research Institute David Geffen School of Medicine University of California, Los Angeles (UCLA) Los Angeles CA

**Keywords:** Countercurrent mechanism, H^+^ transport, thin limb of Henle's loop, vasa recta

## Abstract

SLC4A11 is a multifunctional membrane transporter involved with H^+^ transport, NH_3_ and alkaline pH stimulated H^+^ transport, and water transport. The role of SLC4A11 in the kidney is not well understood. A prior study has shown that in murine kidney, SLC4A11/LacZ staining is primarily in the long‐looped descending thin limb (DTL) as determined by colocalization with aquaporin 1 (AQP1), a protein that is expressed in some, but not all, descending thin limb segments. Using a previously characterized polyclonal antibody, we demonstrate the selective expression of SLC4A11 in the upper DTLs (which are AQP1‐positive) in the outer medulla and inner medulla with little or no expression in the lower DTLs (which are AQP‐1‐null). SLC4A11 also colocalized with AQP1 and the urea transporter UT‐B in the mouse descending vasa recta, but was absent in mouse and rat ascending vasa recta. Mouse, but not rat, outer medullary collecting duct cells also labeled for SLC4A11. Our results are compatible with the hypothesis that in the inner stripe of the outer medulla, SLC4A11 plays a role in the countercurrent transport of ammonia absorbed from the outer medullary thick ascending limb and secreted into the long‐looped DTLs. SLC4A11 can potentially modulate the rate of ammonia transport in the mouse outer medullary collecting duct. Our data suggest functionally unique SLC4A11 pathways in mouse and rat and complement previous studies of DTL Na^+^, urea and water permeability indicating that the upper and lower DTLs of long‐looped nephrons are functionally distinct.

## Introduction

SLC4A11 is a member of the SLC4 bicarbonate (and carbonate) transporter family whose members are expressed in multiple organs and play key roles in systemic acid–base balance, transepithelial transport, intracellular/extracellular ion homeostasis, and cell function (Parker and Boron 2013; Kurtz 2014; Huynh et al. 2018). SLC4A11 (originally called BTR1; (Parker et al. 2001)) was originally reported to transport borate coupled to Na^+^ (Park et al. 2004). SLC4A11 is now thought to be a multifunction transporter that mediates H^+^ flux, NH_3_ and alkaline pH stimulated H^+^ transport, and water flux in the presence of an osmotic gradient (Vilas et al. 2013; Kao et al. 2015, 2016; Zhang et al. 2015; Loganathan et al. 2016; Myers et al. 2016). Unlike other SLC4 transporters that mediate bicarbonate or carbonate transport coupled to Na^+^ and/or Cl^−^, SLC4A11 which is ~35% homologous to other SLC4 transporters is not capable of transporting bicarbonate (or carbonate) (Ogando et al. 2013; Loganathan et al. 2016). In humans, natural mutations in the SLC4A11 gene cause autosomal recessive congenital hereditary endothelial dystrophy (CHED) in the absence or presence of sensorineural deafness (Harboyan syndrome) (Desir et al. 2007; Vithana et al. 2006) in addition to certain cases of Fuchs’ endothelial cell dystrophy (FECD) (Vithana et al. 2008). While no renal phenotype has been reported, patients with mutated SLC4A11 exhibit an anion gap of +68 (with respect to NaCl and KCl), which is compatible with a defect in urine ammonia excretion (Liskova et al. 2015).

The expression of SLC4A11 and its role in the kidney are not well understood. Previous studies in murine kidney have demonstrated differing tubule expression data. In mouse, SLC4A11 expression was localized in DTLs based on LacZ staining (Gröger et al. 2010). In a separate study using an anti‐SLC4A11 antibody, the transporter was localized in the mouse kidney to a region “close to proximal tubules” (Han et al. 2013), whereas in rat using an SLC4A11 antibody, proximal tubules, outer medullary DTLs and inner medullary collecting ducts stained positively (Damkier et al. 2007). In human kidney using the same antibody, SLC4A11 was expressed in the podocytes of the renal corpuscles, on the brush border of proximal tubules, on the apical membrane of intercalated cells in the cortical and outer medullary collecting ducts and on the basolateral membrane of inner medullary collecting ducts (Damkier et al. 2007). None of the previous studies identified SLC4A11 in vasa recta.

In this study, we studied the expression pattern of SLC4A11 in the mouse and rat kidney using a well‐characterized anti‐SLC4A11 polyclonal antibody (Lopez et al. 2009; Kao et al. 2015, 2016). Furthermore, we compared the SLC4A11 expression pattern to AQP1 and urea transporter UT‐B staining. Our results are compatible with the hypothesis that SLC4A11 in the inner stripe portion of the DTL of long‐lopped nephrons can potentially play an important role in renal outer medullary ammonia recycling.

## Methods

### Animals

Adult ICR (CD1) mice were obtained from Envigo (Indianapolis, IN) and mouse tissue sections were obtained from Zyagen (San Diego, CA). Adult Munich Wistar rats were reared in the University Animal Care facility at The University of Arizona (Tucson, AZ). All experiments were conducted in accordance with National Institutes of Health Guide for the Care and Use of Laboratory Animals (1996) and were approved by The University of Arizona Institutional Animal Care and Use Committee.

### Tissue preparation and immunohistochemistry

Kidneys were immersion‐fixed with periodate‐lysine‐paraformaldehyde (PLP) (0.01 mol/L, 0.075 mol/L, 2%) in phosphate‐buffered saline (PBS) (pH 7.4) for 3 h at 4°C, washed in PBS, and dehydrated through an ethanol series. Tissue pieces were embedded in paraffin following ethanol dehydration. Serial transverse tissue sections from defined regions were cut into 4 *μ*m thick sections with a Microm HM 355 S microtome. Immunofluorescence histochemistry was performed as previously described (Wei et al. 2015). Prior to antibody application, paraffin sections were deparaffinized with 3 washes of xylene. Sections were then treated with 0.2% Triton X‐100 (Sigma) in PBS (PBS/Triton) for 2 min and 1% SDS in PBS for 5 min. Thereafter, they underwent three 5‐min PBS/Triton washes and were treated for 10 min with a blocking solution consisting of 5% BSA, 1% normal donkey serum (Jackson ImmunoResearch), and 0.2% Triton X‐100 diluted into PBS. The expression pattern of SLC4A11 in the mouse and rat kidney was assessed using a well‐characterized anti‐SLC4A11 polyclonal antibody raised in rabbit against a 20 amino acid murine C‐terminal peptide (Lopez et al. 2009; Kao et al. 2015, 2016). The tissue was also stained using affinity‐purified polyclonal or monoclonal primary antibodies against water channel aquaporin 1 (AQP1, raised in mouse against human), Abcam #ab9566; water channel aquaporin 2 (AQP2, raised in goat against human), Santa Cruz #9882; urea transporter B (UT‐B, raised in rabbit against rat) provided by Jeff Sands and Janet Klein, Emory University; chloride channel (ClCK, raised in rabbit against rat) and MECA‐32 plasmalemma vesicle‐associated protein (monoclonal antibody raised in rat against mouse), Developmental Studies Hybridoma Bank, University of Iowa. The antibodies were diluted into the blocking solution and then applied for ~12 h at 4°C, followed by three 5‐min PBS/Triton washes. All tubules and blood vessels were labeled nonselectively with fluorescein‐conjugated wheat germ agglutinin (Vector Laboratories #FL‐1021). Secondary antibodies raised in donkey against rabbit, mouse, or goat conjugated to fluorescent probes (Invitrogen/Molecular Probes or Jackson ImmunoResearch) were applied as described previously (Wei et al. 2015). Sections were mounted with Dako fluorescent mounting medium (Carpinteria, CA) and were viewed with epifluorescence microscopy (Applied Precision, DeltaVision). Multiple tissue samples from three‐to‐five male animals were examined for each species and for each region of the medulla.

## Results

### Expression of SLC4A11 in mouse outer medullary long‐looped descending thin limbs (DTLs) and outer medullary collecting ducts (OMCDs)

**Figure 1:**
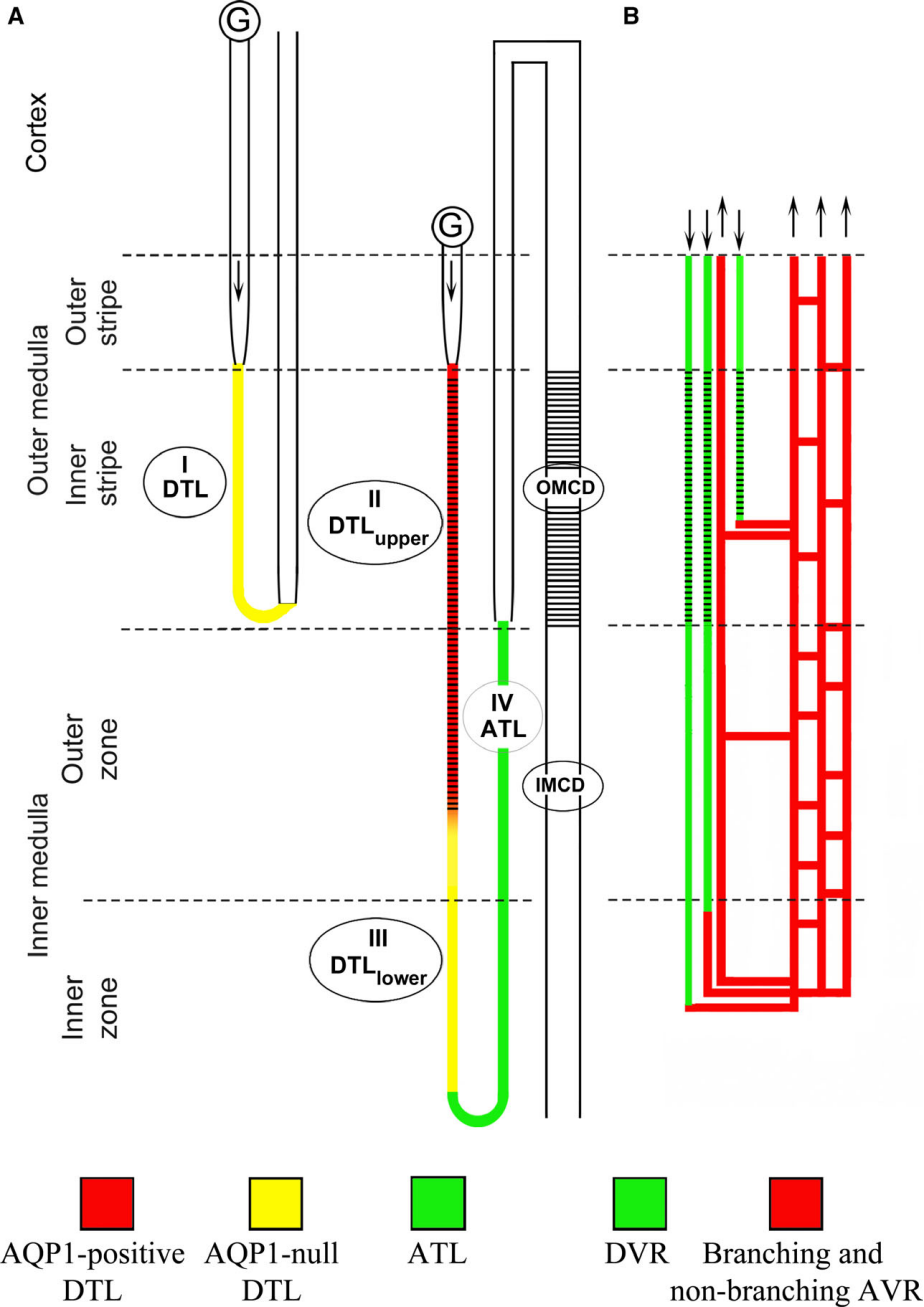
Schematic diagram illustrating the rodent renal medullary nephron segments and blood vessels. (A) Four types of thin limbs of Henle's loops exist in the rodent kidney and include the type I (short‐looped descending thin limb), type II (AQP1‐positive long‐looped descending thin limb or DTL_upper_), type III (AQP1‐null long‐looped descending thin limb or DTL_lower_) and type IV (ascending thin limb). Arrows illustrate flow direction. (B) The renal blood vessels consist of the descending (DVR) and ascending vasa recta (AVR). DVR join a network of branching AVR at any level of the medulla. The branching AVR return plasma to the cortex either directly or by way of the nonbranching AVR that lie in vascular bundles. The expression of SLC4A11 is summarized for the mouse with black cross‐hatches indicating SLC4A11 expression. In contrast to the mouse, little or no SLC4A11 expression was detected in the rat OMCD or DVR.

**Figure 2:**
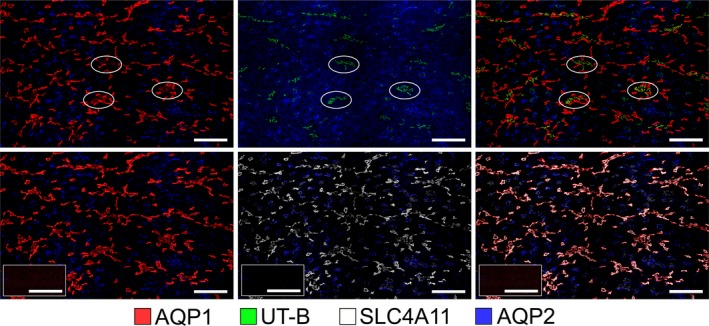
The co‐expression of SLC4A11 with the water channel AQP1 in the mouse ISOM. Tubular SLC4A11 is expressed predominantly in the AQP1‐positive descending thin limbs, which lie both adjacent to and separate from the vascular bundle regions in the ISOM. Vascular bundle regions are identified by the expression of the urea transporter UT‐B in descending vasa recta; 3 bundle regions are circled. OMCDs, which express aquaporin 2 (AQP2), lie only in the interbundle region alongside thick ascending limbs (which are not labeled). Two serial transverse sections (sections 1 (top) and 2 (bottom) are shown. A third serial section (insets, bottom row) was treated identically with the absence of primary antibodies (left, TRITC+DAPI; middle, FITC+DAPI; right, TRITC+FITC+DAPI). Scale bars, 100 *μ*m.

A schematic diagram of the long‐looped descending thin limb (which consists of an upper and a lower DTL segment), ascending thin limb and vasa recta are shown in Figure 1. AQP1 is expressed in the upper portions of descending segments of rodent long‐looped nephrons that pass through the inner stripe of the outer medulla (ISOM) and outer inner medulla (IM) (“upper DTLs”) (Dantzler et al. 2014; Nawata et al. 2014) (Fig. 2). The “lower DTL” segments, which are also present in both the outer medulla (OM) and IM, do not express detectable AQP1 and include both long‐looped DTLs and descending segments that have been recently defined in the mouse as the intermediate loop DTLs (Kim et al. 2016).

There are no reported antibodies that serve as selective markers for the AQP1‐null lower DTLs in rat or mouse. As shown in Figure 2, all of the SLC4A11‐positive thin limb segments in the OM colocalize with AQP1. Therefore, SLC4A11 is expressed along the AQP1‐positive segment of the outer medullary DTL and is not expressed in the AQP1‐null segments of DTLs that extend within and beyond the ISOM in the IM. Tissue sections treated without the primary antibody showed no nonspecific label from the secondary antibody (Fig. 2).

The rodent ISOM consists of two regions in the transverse dimension – the bundle and interbundle regions. Descending vasa recta (DVR) are located within the bundle regions (Fig. 2) along with the ascending vasa recta (AVR) and short‐looped DTLs (both of which are not shown). The interbundle region consists of the long‐looped DTLs, medullary thick ascending limbs (mTALs), OMCDs, and a network of branching ascending vasa recta. In the mouse, SLC4A11‐positive and AQP1‐positive DTLs tend to be clustered adjacent to the bundle region, but also lie more centrally within the interbundle region (Fig. 2).

**Figure 3:**
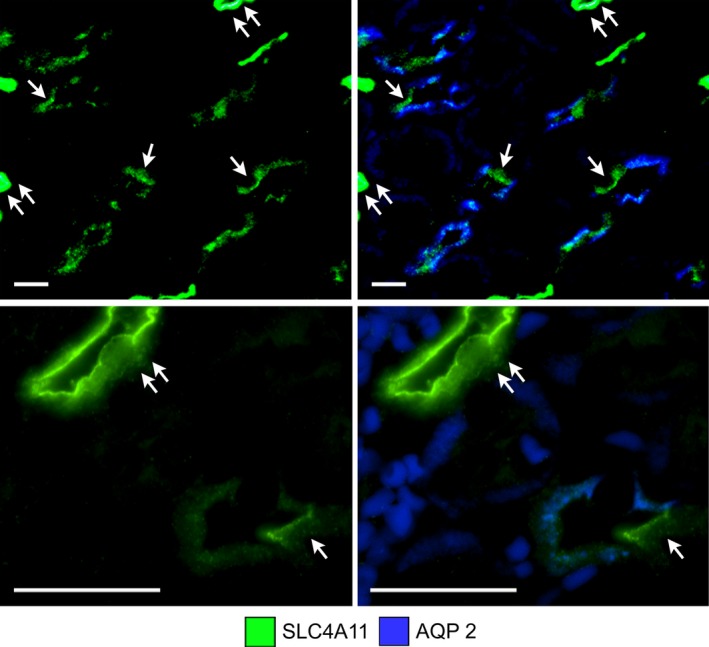
SLC4A11 expression in two transverse sections of the ISOM (low power, upper images; high power, lower images) without and with AQP2 expression. SLC4A11 is expressed in most cells in mouse OMCDs, including the intercalated cells (single arrows) that lie in the interbundle region (upper panels). SLC4A11 is expressed most heavily in the apical membrane of OMCDs and descending thin limbs. The descending thin limbs are marked with double arrows. The descending thin limb in the lower panels is sliced tangentially. Scale bars, 20 *μ*m.

SLC4A11 expression is more predominant in the DTL apical membrane compared to the basolateral membrane (Fig. 3). SLC4A11 is also expressed in cells of the OMCD in the ISOM, including principal and intercalated cells; the latter are identified by their lack of AQP2 expression (Fig. 3). In these cells, SLC4A11 is predominantly apical.

### Expression of SLC4A11 in mouse outer medullary descending vasa recta (DVR)

**Figure 4:**
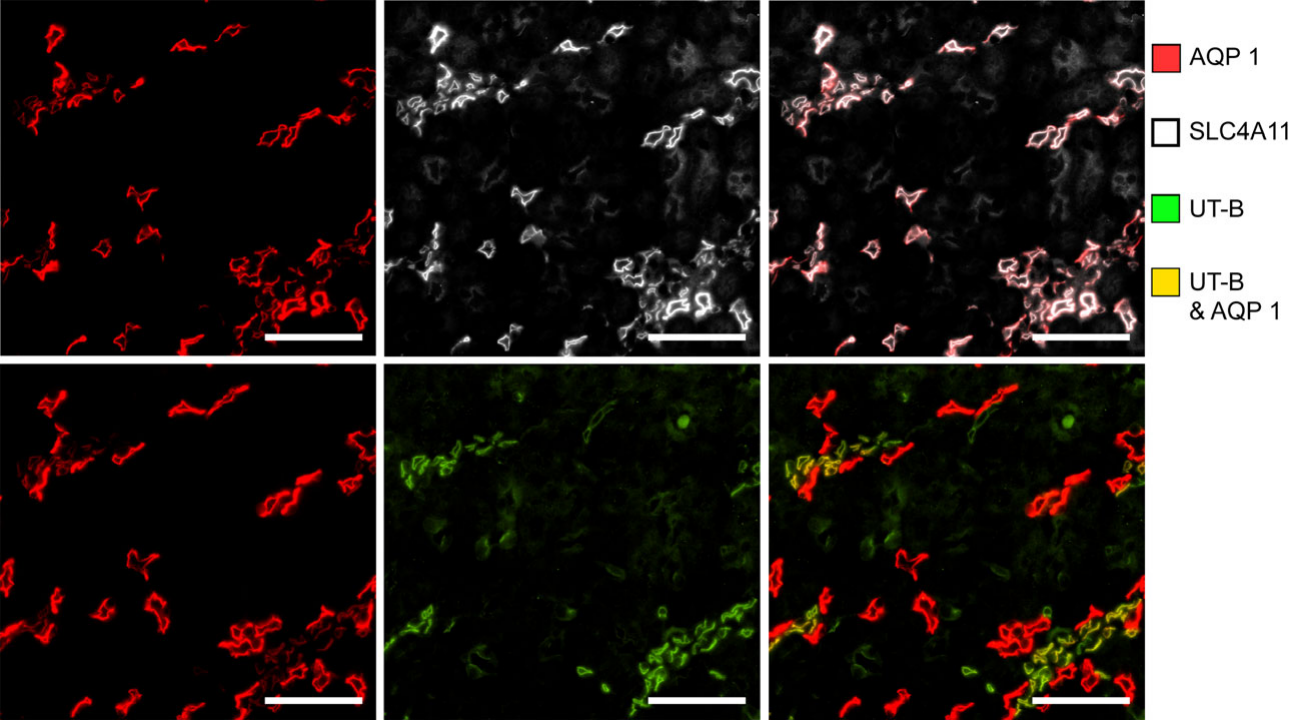
SLC4A11 is expressed in the mouse descending vasa recta as shown in a transverse section of the ISOM. Descending vasa recta are identified by expression of the urea transporter UT‐B. Descending vasa recta also express AQP1 but at a lower intensity level than descending thin limbs. Scale bars, 50 *μ*m.

The urea transporter UT‐B is selectively expressed in DVR, and AQP1 is expressed in some of these vascular segments. As shown in Figure 4, SLC4A11 is co‐expressed with UT‐B and AQP1 in the mouse DVR. The endothelium of DVR is thinner than the epithelium of DTLs; therefore, SLC4A11 and AQP1 expression appear weaker in DVR compared to DTLs (Fig. 4).

**Figure 5:**
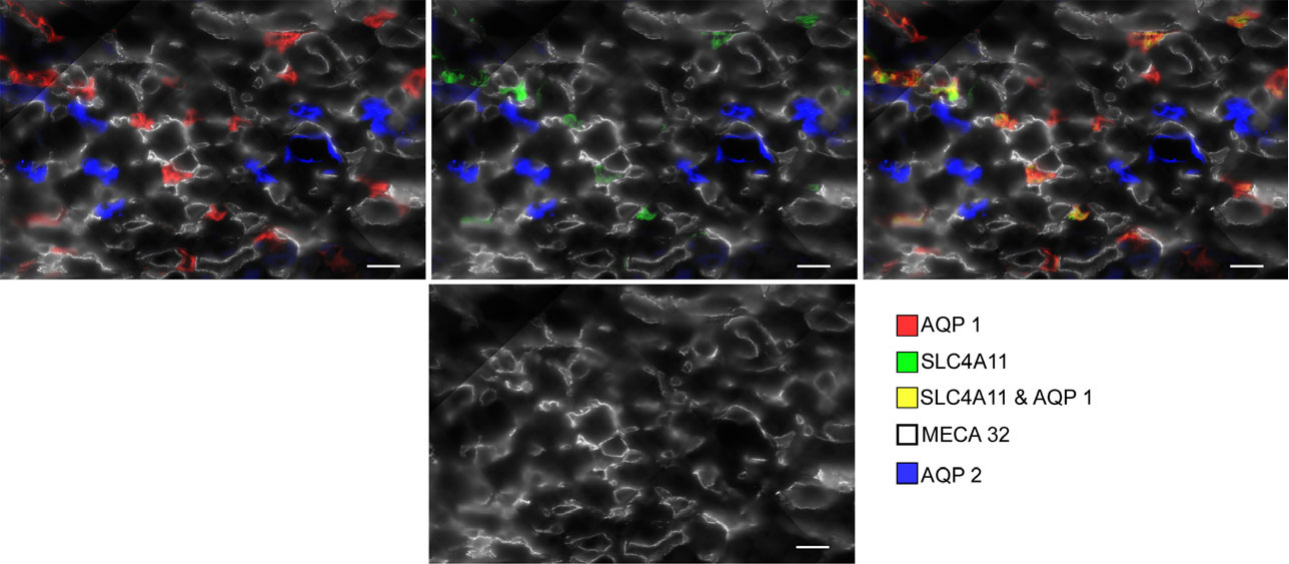
SLC4A11 is not expressed in mouse ascending vasa recta as shown in a transverse section through the ISOM interbundle region. MECA 32 labels plasmalemma vesicle‐associated protein and is specific for ascending vasa recta. AQP1 is also not expressed in ascending vasa recta. Scale bars, 20 *μ*m.

The endothelial plasmalemma vesicle‐associated protein (PV1, PLVAP) is a marker of fenestrated capillaries in the medulla. In the kidney, all medullary ascending vasa recta, including the branching ascending vasa recta are fenestrated and express PV1; however, these vessels do not express detectable AQP1 or SLC4A11 (Fig. 5).

### Expression of SLC4A11 in the cortex and outer stripe of the mouse outer medulla

**Figure 6:**
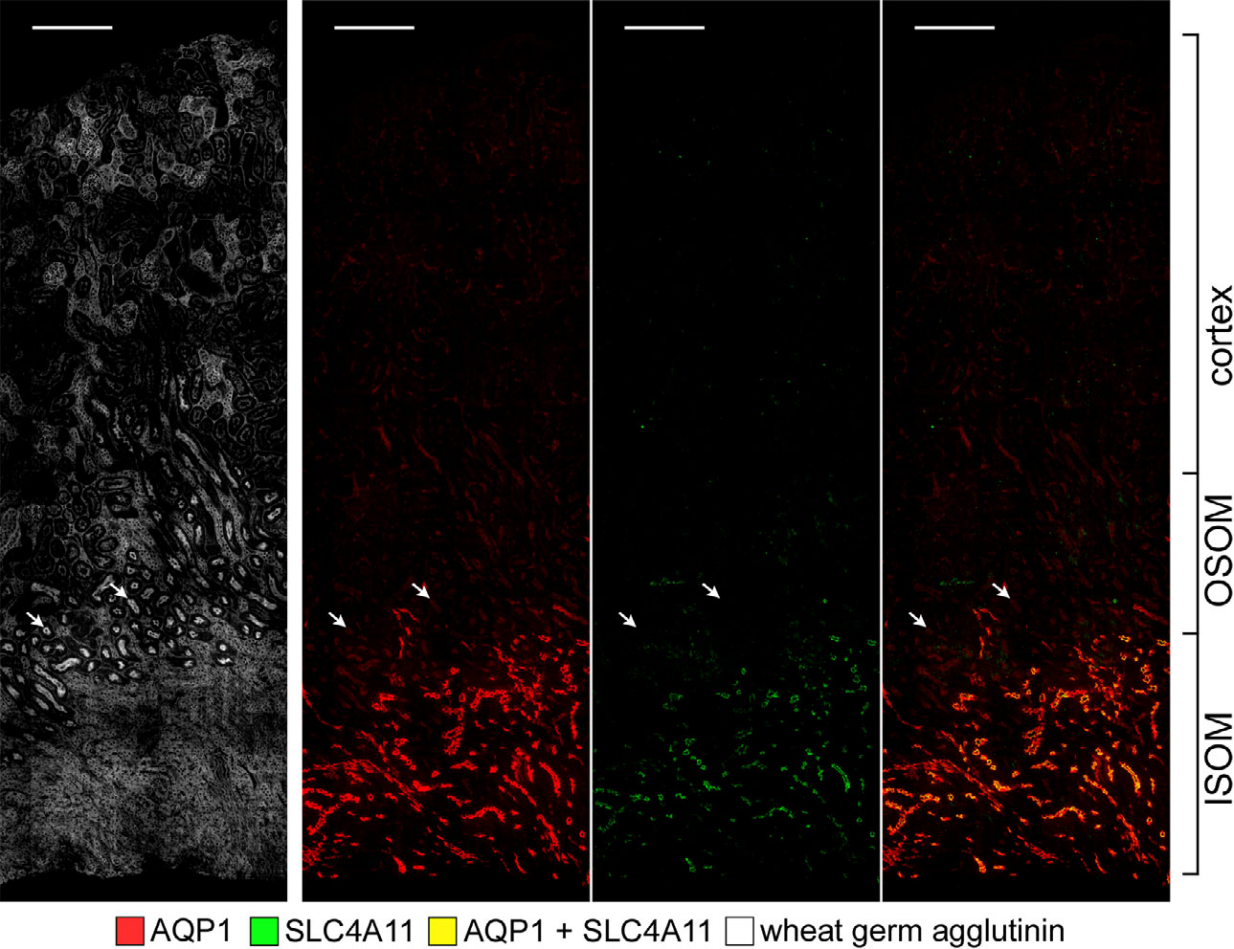
Expression of SLC4A11 in the long‐looped descending thin limbs in a longitudinal section through the cortex, OSOM and ISOM. All tubules are labeled with wheat germ agglutinin. Weak or no SLC4A11 expression is seen in proximal tubules (arrows) relative to the abundant expression in descending thin limbs. Scale bars, 200 *μ*m.

In the mouse, whereas SLC4A11 is abundantly expressed in the ISOM, relatively lower or no expression was observed in the nephron segments of the cortex and OSOM (Fig. 6).

### Expression of SLC4A11 in mouse inner medullary long‐looped DTLs

**Figure 7:**
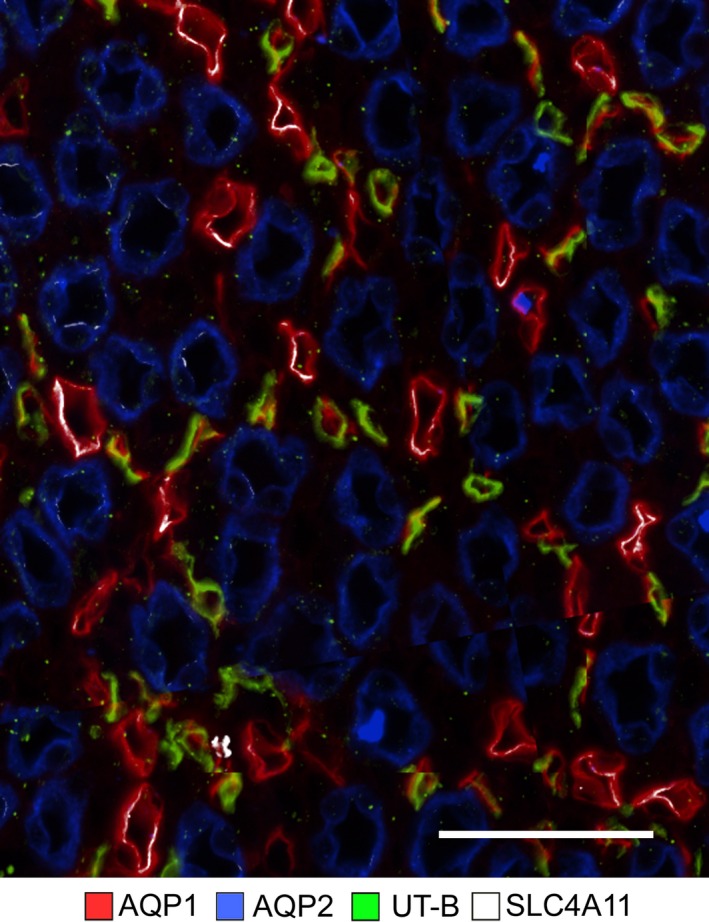
In the mouse inner medullary thin limbs of Henle's loops, SLC4A11 is expressed predominantly in AQP1‐positive segments. The figure consists of a composite overlay of two transverse sections from the upper 30% of the inner medulla. Little or no SCL4A11 is expressed in AQP1‐null or UT‐B‐positive segments. Scale bar, 50 *μ*m.

The inner medullary SLC4A11 expression for the mouse is shown in Figure 7. SLC4A11 colocalizes with AQP1 in all AQP1‐positive DTL segments. The AQP1‐null segments, DVR (UTB‐positive segments), and IMCDs do not express detectable SLC4A11.

### Expression of SLC4A11 in rat outer medullary long‐looped DTLs

**Figure 8:**
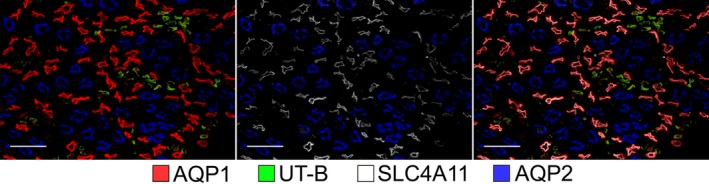
The co‐expression of SLC4A11 with the water channel AQP1 in the rat ISOM. Tubular SLC4A11 is expressed predominantly in the AQP1‐positive descending thin limbs. Vascular bundle regions are identified by the expression of the urea transporter UT‐B in descending vasa recta. OMCDs, which express aquaporin 2 (AQP2), lie only in the interbundle region alongside thick ascending limbs (which are not labeled). The figure consists of a composite overlay of two transverse sections. Scale bars, 100 *μ*m.

AQP1 is expressed in the upper portions of the descending segments of rat long‐looped nephrons (“upper DTLs”) (Fig. 8). The “lower DTL” segments do not express detectable AQP1. As shown in Figure 8, all of the SLC4A11‐positive segments in the rat OM colocalize with AQP1. Therefore, in the rat, SLC4A11 is expressed primarily along the AQP1‐positive segment of the outer medullary DTL and is not expressed in the AQP1‐null segments of DTLs nor in the DVR or OMCD.

### Expression of SLC4A11 in rat inner medullary long‐looped DTLs

**Figure 9:**
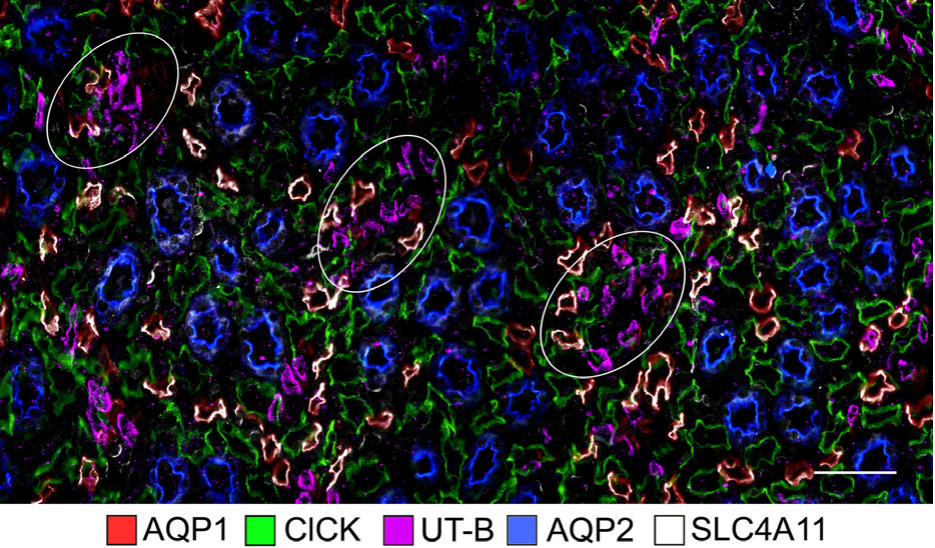
In the rat inner medullary thin limbs of Henle's loops, SLC4A11 is expressed predominantly in AQP1‐positive segments, and little or no expression occurs in the AQP1‐null descending thin limbs, ClCK‐positive segments (ascending thin limbs), UT‐B‐positive vasa recta (descending vasa recta) or in the IMCD. Three vascular bundles in which descending vasa recta predominate are circled. The image consists of a composite overlay of three serial transverse sections from the upper 30% of the inner medulla. ClCK expression in ascending thin limbs indicates the approximate total number of thin limbs in transverse sections. Scale bar, 50 *μ*m.

The SLC4A11 expression in a tissue section from the upper 30% of the rat IM is shown in Figure 9. SLC4A11 colocalizes with AQP1 in all segments. The AQP1‐null segments, DVR (UTB‐positive segments) and IMCDs do not express detectable SLC4A11. The absence of SLC4A11 expression in the AQP1‐null segment is further illustrated for the rat IM, in which all ATLs were selectively labeled using an antibody against the Cl channel ClCK. 2‐5% of the ClCK‐positive segments are known as prebend descending segments, which extend along the terminal ~200 *μ*m of the DTL (Pannabecker 2008). Assuming equivalence of descending and ascending segments in any random tissue section, the ratio of AQP1‐positive‐to‐AQP1‐null DTLs in this section is approximately 4:3. We are unaware of an antibody that selectively labels mouse ATLs.

The expression of SLC4A11 protein along the tubular and vascular segments of the mouse kidney is summarized schematically in Figure 1. SLC4A11 is expressed predominantly in the DTL_upper_ and outer medullary DVR. A very weak expression was observed in cells of some, but not all, OMCD segments in the mouse. Little or no SLC4A11 expression was detected in the rat OMCD or DVR.

## Discussion

Our new data on SLC4A11 expression in the kidney complement previous studies of DTL Na^+^, urea and water permeability indicating that the upper and lower DTLs of long‐looped nephrons are functionally distinct (Nawata et al. 2014). In previous studies, we have documented the complex heterogeneity of AQP1 and UT‐B expression in the rodent OM. In most rodent and human short‐looped DTLs, little or no AQP1 is expressed along the entire length (Zhai et al. 2007). In the DVR, AQP1 and the urea transporter UT‐B are expressed along the entire length (Nawata and Pannabecker 2018). SLC4A11 is expressed in AQP1‐positive DTLs in the mouse and rat OM and IM. The absolute colocalization of SLC4A11 with AQP1 confirms the absence of SLC4A11 in the AQP1‐null DTL. This absence is clearly illustrated for the rat IM, in which the relative abundance of AQP1‐positive and AQP1‐null DTLs can be determined by comparison to the abundance of ascending thin limbs. SLC4A11 colocalized with AQP1 and UT‐B in the outer medullary DVR in the mouse but not rat, whereas no SLC4A11 was detected in the inner medullary DVR nor in the AVR in either species. SLC4A11 staining was also detected in the OMCD principal and intercalated cells of the mouse but not rat, and no SLC4A11 was detected in the upper 30% of the rat or mouse inner medulla. Relatively few intercalated cells occur in this region of the collecting duct.

Two previous studies using immunohistochemistry have reported expression of SLC4A11 in additional nephron segments of the mouse, rat, and human kidney compared to those that we have identified (Damkier et al. 2007; Gröger et al. 2010). These include the glomerular podocytes and cortical proximal tubules in the human and the glomeruli, proximal tubules of the outer stripe of the OM and IMCD of the mouse or rat kidneys. The differences between those two studies and ours may reflect, at least in part, both qualitative and quantitative differences that arise from variable detectability levels. In the previous rat study, SLC4A11 tissue gene expression increased gradually from cortex to the outer medulla to the inner medulla; however, for the rat and human, protein expression was only shown in the principal cells of the IMCD from the deep papilla (Damkier et al. 2007). In the latter study, the papillary IMCD can be identified by their large diameter and by the sparse double‐layered epithelium. We speculate that, for the mouse, rat and human, while SLC4A11 protein expression occurs in the cortical collecting ducts and OMCD, it is expressed relatively more abundantly in collecting ducts of the lower inner medulla (and only in principal cells). Our study did not investigate this region of the medulla. SLC4A11 protein expression differences potentially play a role in variable solute transepithelial transport and countercurrent mechanisms between the three species.

Recent studies have provided strong evidence that SLC4A11 functions as an ammonia and alkaline pH stimulated H^+^ transporter and that Na^+^, Cl^‐^, and K^+^ are not transported ions. This finding coupled with our new expression data are compatible with the hypothesis that in the upper DTL and initial DVR, SLC4A11 plays a potential role in the countercurrent transport of ammonia absorbed from the outer medullary thick ascending limb. Furthermore, in the OMCD (inner stripe), apical SLC4A11 may modulate the rate of luminal ammonia secretion.

**Figure 10:**
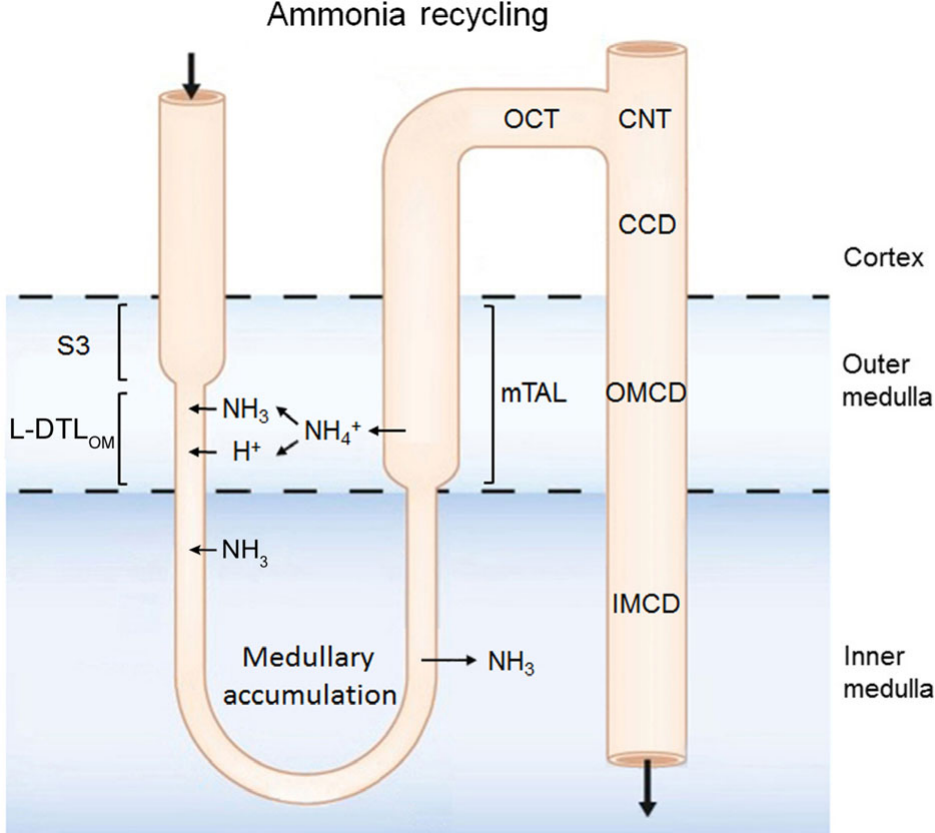
Model of countercurrent transport of ammonia in the outer medulla. Ammonia absorbed by the mTAL is recycled either into the S3 portion of the proximal tubule and descending thin limb of the loop of Henle, a process that plays an important role in generating the corticomedullary ammonia concentration gradient. In addition, a portion of the ammonia absorbed into the interstitium by the mTAL is secreted into the lumen of the outer medullary collecting duct. The expression of SLC4A11 in the descending thin limb of long‐looped nephrons provides a pathway for ammonia recycling in this segment. DTL_OM_, long‐looped descending thin limb of the outer medulla.

In the mammalian kidney, the ability to produce a concentrated urine is associated with the generation of a corticomedullary osmolality gradient (increasing from cortex to medullary tip) (Dantzler et al. 2014). The underlying processes that generate this gradient differ in the outer versus the inner medulla. In the outer medulla, it is generally believed that there is a countercurrent‐generating process resulting from active transport of NaCl by the mTAL. This segment has a very low water permeability (Rocha and Kokko 1973) and its active transport processes increase the peritubular osmolality of the anatomically adjacent long‐looped DTLs in the OM (Kriz et al. 1972). These tubules express AQP1 and have a high water permeability unlike short‐loop descending thin limbs (Kim et al. 2016). Previous studies have also provided support for a countercurrent ammonia recycling system in the OM. In this scheme, a portion of the ammonia absorbed by the mTAL is coupled to ammonia secretion by the DTL of long‐looped nephrons in the OM (Fig. 10). Of the tubule components involved in outer medullary ammonia recycling, the transport processes mediating mTAL ammonia absorption (and their regulation) are well understood (By et al. 1994; Mount 2014). The luminal absorption of ammonia via NKCC2 (apical) and basolateral secretion via NHE4 ensures that the luminal and peritubular fluid composition in the more distal nephron segments favors normal tubule ammonia secretion in the cortical collecting duct, OMCD, and IMCD (Weiner and Verlander 2013, 2014) with subsequent urinary ammonia excretion. The delivery of ammonia to the IM via the descending limb of long‐loop nephrons is required for generating the corticomedullary ammonia gradient (Good et al. 1987; Garvin et al. 1988; Flessner et al. 1993)*;* a process which is modulated in response to metabolic acidosis (Stern et al. 1985). Despite the importance of DTL ammonia secretion in this model, there has been no progress in identifying potential transport pathways involved (Tabei and Imai 1987; Flessner et al. 1993). Our study is the first to identify SLC4A11 as a candidate protein based on its expression profile in the mouse renal medulla.

Two studies that have reported urine data in mice with loss of SLC4A11 have reported discrepant results. In the study of Gröger et al. (2010) that showed DTL expression by LacZ staining, the mice had decreased urine osmolarity and increased Na^+^ and water excretion that was hypothesized to be due to impaired DTL Na^+^ transport. Han et al. (2013) also reported a decrease in urine osmolality; however, the urine Na^+^ concentration in addition to K^+^, Cl, Ca^2+^, and Mg^2+^ concentration was decreased (without reporting excretion rates per se). Whether differences in mice background strains account for these findings is unclear. Furthermore, the effects of water restriction remain untested; therefore, the maximal urine concentrating ability of the SLC4A11‐null mice is unknown. Although SLC4A11 can also transport water in the presence of an osmotic gradient, given the contribution of AQP1 to DTL water transport, it is unlikely that loss of SLC4A11 plays an important role in this regard. However, it is possible considering the colocolization of SLC4A11, AQP1, and UT‐B documented in our study that mice lacking SLC4A11 down‐regulate AQP1 and/or UT‐B and therefore the primary cause of the reported urine chemistry changes needs further consideration. In neither study was ammonia excretion nor urine acid–base parameters measured. In vitro tubule perfusion studies have shown that the upper portion of the descending segment of long‐looped nephrons has a high ammonia permeability and passive whole tubule ammonia secretion rate (Flessner et al. 1993), and therefore it would be important in future studies to compare these parameters in wild‐type mice versus animals with loss of the transporter.

In summary, in the this study, we demonstrated the expression profile of SLC4A11 in the mouse kidney. Based on its expression profile, we postulate that the transporter is a candidate protein in the DTL for mediating outer medullary ammonia secretion and countercurrent recycling, thereby maintaining the cortical medullary ammonia gradient and collecting duct ammonia secretion. Expression of SLC4A11 in the outer medullary descending vasa recta and their absence in ascending vasa recta suggests that selective and/or mediated ammonia flux and delivery by medullary blood vessels may provide an additional pathway in an ammonia recycling mechanism.

## Conflict of Interest

None declared.
